# Microcarriers encapsulating COL1A1 mRNA-loaded nanovesicles for skin photoaging treatment

**DOI:** 10.1016/j.mtbio.2026.103126

**Published:** 2026-04-15

**Authors:** Xiang Lin, Anne M. Filppula, Luoran Shang, Hongbo Zhang, Dexuan Wang

**Affiliations:** a Department of Pediatrics, The Second Affiliated Hospital and Yuying Children's Hospital of Wenzhou Medical University, Wenzhou, 325027, China; b Pharmaceutical Sciences Laboratory, Department of Natural and Health Sciences, Åbo Akademi University, Turku, 20520, Finland; c Shanghai Xuhui Central Hospital, Zhongshan-Xuhui Hospital, and the Shanghai Key Laboratory of Medical Epigenetics, International Co-laboratory of Medical Epigenetics and Metabolism (Ministry of Science and Technology, Institutes of Biomedical Sciences), Fudan University, Shanghai, 200032, China

**Keywords:** Nanovesicles, Microfluidic electrospray, mRNA delivery, Microcarriers, Photo-aging

## Abstract

Extracellular vesicles have shown great potential in treating ultraviolet (UV)-induced skin photoaging. However, effective delivery of these bioactive agents to achieve long-term therapeutic effects remains a significant challenge. In this study, we have developed recombinant human collagen (RHC)-based microcarriers loaded with native exosomes as well as Collagen type I alpha 1 chain (COL1A1) mRNA-encapsulating nanovesicles (Emvs) to treat skin photoaging. The microcarriers mitigated UV-induced cellular senescence in HaCaT cells, as evidenced by reduced oxidative stress, decreased apoptosis, and attenuation of senescence-associated markers. In the animal model, the microcarriers not only attenuated wrinkle formation, but also promoted type I collagen deposition in UV-damaged skin. These results together with the demonstrated biocompatibility highlight the potential of the microcarrier delivery system in photoaging treatment.

## Graphical abstract

**Figure:**
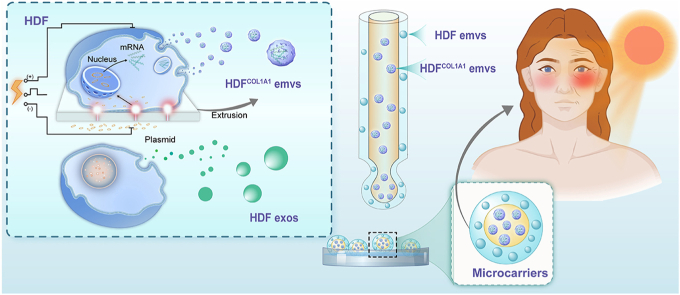

## 1 Introduction

Ultraviolet (UV) radiation-induced photoaging represents a major form of extrinsic skin aging, manifested by collagen degradation, elastin fiber accumulation, and wrinkle formation [[1], [2], [3], [4]]. Chronic UV exposure induces excessive oxidative stress and DNA damage. These processes accelerate extracellular matrix (ECM) degradation, particularly collagen loss, thereby compromising skin elasticity and structural integrity [[5], [6], [7]]. This can result in skin wrinkling, loss of firmness, and other visible signs of aging [5,7,8]. Current mainstay treatments for photoaging include topical antioxidants, retinoids, and other skincare formulations [[9], [10], [11], [12], [13]]. However, these approaches tend to offer limited benefits, particularly in restoring collagen. More advanced biological agents, such as nanovesicles, have been explored to enhance skin regeneration [[14], [15], [16], [17]]. However, direct administration of nanovesicles often suffer from poor viability and side effects, leading to inconsistent therapeutic outcomes. Although biomaterials such as hydrogels have been utilized to encapsulate and deliver various bioactive agents, current delivery systems still fall short of providing comprehensive treatment at different stages of photoaging [[18], [19], [20], [21], [22]]. Therefore, the development of on-demand drug delivery strategies for anti-aging is crucial.

**Fig. 1:**
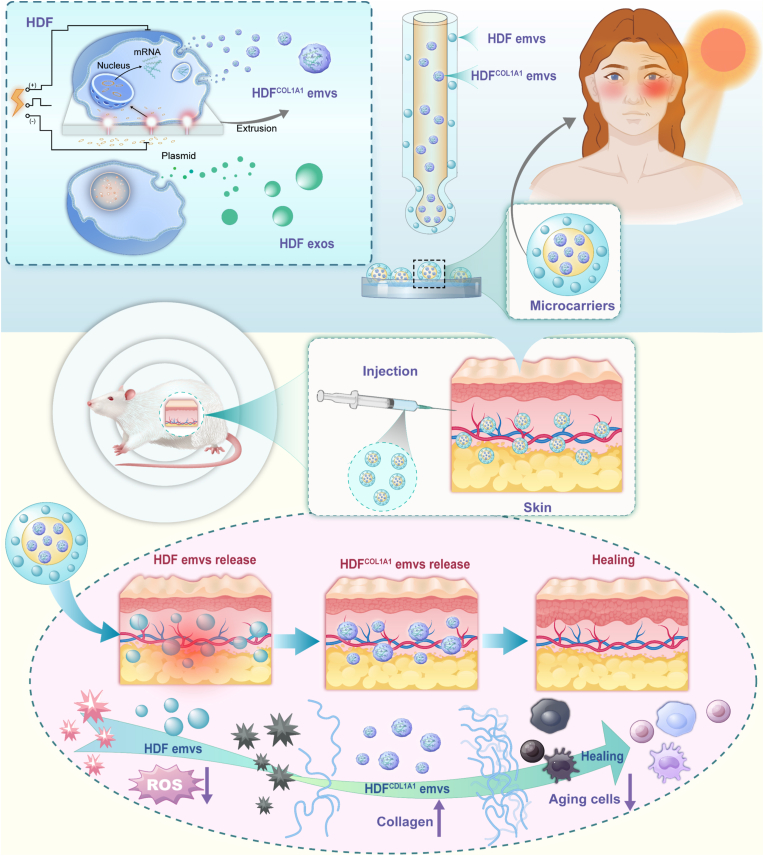
Scheme of the fabrication of the microcarriers and delivery of engineered nanovesicles for photoaging therapy. Abbreviations: HDF, human dermal fibroblast; Exos, exosomes; Emvs, engineered nanovesicles; HDF^COL1A1^ Emvs, Emvs derived from HDFs transfected with COL1A1 plasmid (COL1A1 mRNA–encapsulating nanovesicles); ROS, reactive oxygen species; COL1A1, collagen type I alpha 1 chain.

In this study, we introduce recombinant human collagen (RHC) microcarriers, incorporating engineered exosome-mimetic nanovesicles (Emvs) for the treatment of UV-induced skin photoaging. Messenger RNA (mRNA) therapy is an emerging biotechnology that uses synthetic mRNA to directly instruct cells to produce specific proteins (Fig. 1) [[23], [24], [25]]. The core principle of this approach in the delivery of target-encoding mRNA into cells, where the cellular translation machinery synthesizes the desired protein [[26], [27], [28], [29]]. Nevertheless, mRNA-based approaches for skin regeneration remain challenged by poor stability, limited local retention, and inefficient delivery, underscoring the need for advanced carrier systems. The efficacy of this treatment depends largely on the effectiveness of the delivery system, which must ensure that the genetic information is safely, efficiently, and stably translated into functional proteins. To address this challenge, the microfluidic electrospray approach enables fabrication of microcarriers for precise accommodation of delivery vehicles, allowing for controlled structuring, optimized drug-loading capacity, and tunable release kinetics [[30], [31], [32], [33], [34]]. Thus, it is feasible to envision a smart delivery system wherein engineered nanovesicles carrying desired mRNA could provide prolonged, effective treatment for photoaged skin.

Here, human dermal fibroblasts (HDFs) were first electroporated with a plasmid encoding collagen type I alpha 1 (COL1A1) fused with GFP, followed by extrusion to generate Emvs enriched with COL1A1 mRNA. The designed microcarriers were fabricated via microfluidic electrospray, with a recombinant human collagen (RHC) core encapsulating the Emvs enriched with COL1A1 mRNA and an alginate shell loaded with untransfected Emvs. In HaCaT cell assays, the microcarriers effectively alleviated UVB-induced inflammation, as demonstrated by reduced levels of reactive oxygen species (ROS) and decreased apoptosis. *In vivo*, subcutaneous injection of the microcarriers helped to establish a favorable microenvironment. In parallel, the gradual release of Emvs enriched with COL1A1 mRNA contributed to enhanced type I collagen deposition and tissue remodeling in UVB-damaged skin. This combinatory strategy serves as a valuable intervention for the management of photoaging, providing prolonged and comprehensive therapeutic effects for UVB-damaged skin.

## 2 Results and discussion

### 2.1 Emvs preparation

**Fig. 2:**
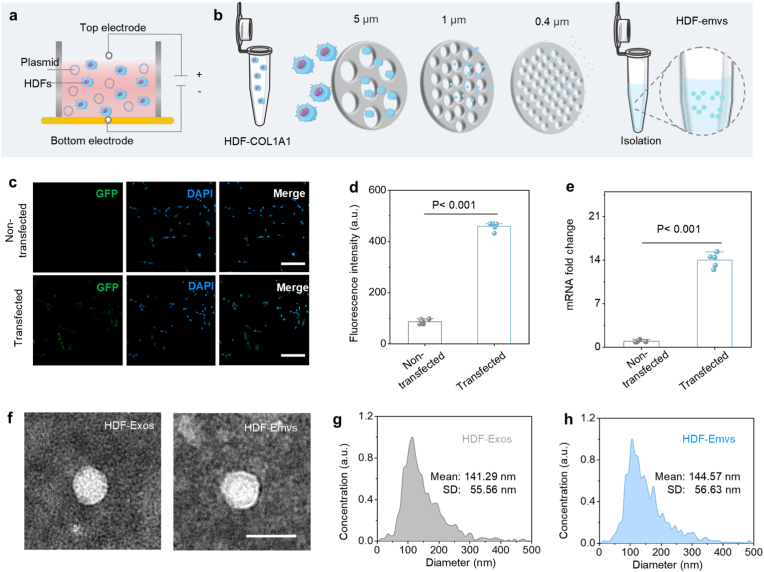
**Plasmid transfection and Emvs preparation**. a) Schematic illustration of the electroporation process. b) Schematic of Emvs preparation using the extrusion method. c) Immunofluorescence staining of transfected and non-transfected HDF cells, with green indicating the plasmid and blue indicating DAPI. d) Quantification of fluorescence intensity (n = 5). e) RT-qPCR of COL1A1 mRNA in Ctrl (non-transfected) and transfected groups in 24 h (n = 5). f) TEM images of HDF-Exos and HDF-Emvs. g) Size distribution of HDF-Exos. h) Size distribution of HDF-Emvs. The scale bar is 50 μm in c), 100 nm in f). (For interpretation of the references to color in this figure legend, the reader is referred to the Web version of this article.)

To generate vesicles enriched with COL1A1 mRNA, primary human dermal fibroblasts (HDFs) were first electroporated with a COL1A1–GFP expression plasmid (Fig. 2a). Extrusion-derived membrane vesicles (Emvs) were subsequently harvested from COL1A1-transfected HDFs using the LiposoFast LF-50 extruder (Fig. 2b). Naturally secreted extracellular vesicles (Exos) were isolated via ultracentrifugation. Successful transfection was confirmed by immunofluorescence imaging and RT–qPCR analysis, which demonstrated increased intracellular expression of COL1A1 mRNA in transfected HDFs (Fig. 2c–e). Western blot analysis detected typical vesicle-associated proteins Alix, CD63, and TSG101 in both Emvs and Exos. Notably, the endoplasmic reticulum marker Calnexin was detectable in Emvs but largely absent in Exos, reflecting differences in membrane origin and biogenesis between extrusion-derived vesicles and naturally secreted exosomes (Fig. S1). Transmission electron microscopy revealed characteristic cup-shaped vesicle morphology for both HDF-derived Emvs and Exos (Fig. 2f), with size distributions shown in Fig. 2g and h. These results confirm the successful generation of COL1A1 mRNA–enriched Emvs and establish a robust experimental basis for comparing their cargo loading efficiency and functional delivery potential with naturally secreted exosomes.

COL1A1 mRNA in different vesicles was quantitatively analyzed by RT-qPCR. Compared to non-transfected Emvs, transfected Emvs exhibited a significantly higher COL1A1 mRNA level (Fig. S2). Consistent with this, the total protein content and particle number of HDF-Emvs were higher than those of HDF-Exos (Fig. S3). Stability experiments showed that HDF-Emvs remained stable at room temperature and under serum-containing conditions at 4 °C (Fig. S4a). To assess functional delivery, untransfected HDF cells were treated with Emvs loaded with COL1A1 for 72 h, and the results showed a concentration-dependent increase in cell proliferation (Fig. S4b). Correspondingly, Western blotting confirmed increased COL1A1 protein expression in recipient HDF cells (Fig. S4c). The successful translation of COL1A1 protein in recipient cells further indicates that vesicle-associated mRNA is functionally delivered. In summary, these results demonstrate the successful preparation of COL1A1-loaded Emvs, highlighting their superior mRNA loading capacity and functional delivery performance compared to naturally secreted exosomes.

### 2.2 Microcarrier fabrication and characterization

**Fig. 3:**
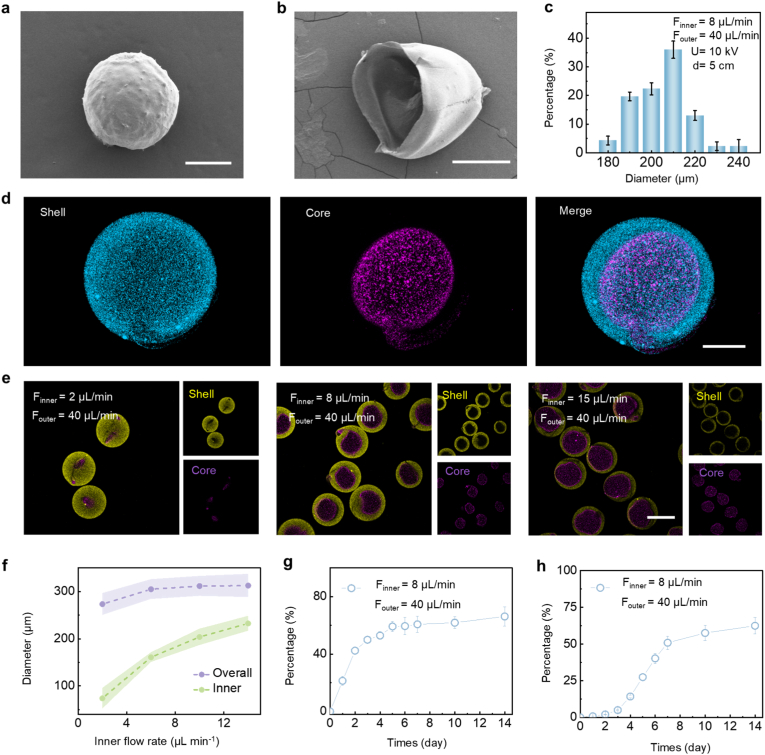
**Microcarrier characterization**. a) Representative SEM image of a microcarrier. b) Representative SEM image of the cross-sectioned view of a microcarrier (n = 3). c) Particle size distribution. d) Fluorescence images illustrating the localization of nanoparticles within a representative microcarrier. Fluorescent nanoparticles in the shell were used to simulate un-transfected Emvs, while nanoparticles in the core were used to simulate Emvs. e) Fluorescence images illustrating the localization of nanoparticles in microcarriers of various sizes. f) Impact of inner phase flow rates on the core diameter and overall diameter of the microcarriers (n = 3). g) Release profile of nanoparticles from the shell layer. The microcarrier contains nanoparticles only in the shell layer (n = 3). h) Release profile of nanoparticles from the core (n = 3). The microcarrier contains nanoparticles only within the core. Scale bars: 100 μm (a–b, d); 300 μm (e).

Recombinant human collagen methacryloyl (RHCMA) was selected as the core matrix due to its excellent biocompatibility, intrinsic relevance to skin extracellular matrix, and ability to provide a supportive microenvironment for fibroblast function. In addition, the photo-crosslinkable RHCMA network enables stable encapsulation and sustained release of bioactive nanovesicles. Sodium alginate was chosen as the shell material because of its mild gelation via Ca^2+^ crosslinking and permeability, which allows rapid initial release while maintaining overall structural stability. To generate core-shell microcarriers, a capillary-based microfluidic electrospray device was assembled with two coaxial capillaries on a glass slide (Fig. S5a–b). The inner phase fluid contained photo-crosslinkable RHCMA solution with LAP, and the outer phase was a 2% sodium alginate solution. Under an external electric field, the liquids fragmented into droplets at the tip. The droplets were collected in 2% CaCl_2_ solution, where the alginate shell rapidly crosslinked, followed by UV curing of the core. Scanning electron microscopy (SEM) imaging confirmed the spherical shape and core-shell structure of the microcarriers (Fig. 3a–c).

To visualize cargo distribution within the core–shell structure, two fluorescent nanoparticles with distinct emission colors were used as model cargos. The corresponding fluorescence images demonstrated uniform nanoparticle distribution within the core and shell layers of the microcarriers (Fig. 3d and e). To quantify vesicle loading efficiency, Emvs were encapsulated into the alginate shell and RHCMA core, respectively. The encapsulation efficiency of Emvs loaded into the alginate shell was 48.2 ± 3.1%, whereas Emvs within the core exhibited a significantly higher encapsulation efficiency of 82.6 ± 2.4%, likely due to the denser photo-crosslinked RHCMA network. We further analyzed factors affecting microcarrier size, including collection distance and applied voltage (Fig. S6–b). Microcarrier diameter increased with increasing collection distance but decreased with increasing voltage. Within the tested range, the inner phase flow rate mainly influenced the core size, while the outer phase flow rate affected both shell thickness and overall diameter (Fig. 3f and S6c). To evaluate release behavior, microcarriers containing nanoparticles only in the core or shell were immersed in PBS, and fluorescence intensity was monitored over time. This design allowed separate analysis of the release profiles from each layer. Notably, nanoparticles encapsulated in the core exhibited a significantly slower release rate than those in the shell (Fig. 3g and h). These results demonstrate that the microfluidic electrospray approach enables precise control over microcarrier architecture, vesicle loading efficiency, and release kinetics, providing a robust platform for partitioned and sustained delivery of bioactive nanovesicles.

### 2.3 *In vitro anti*-photoaging effect

**Fig. 4:**
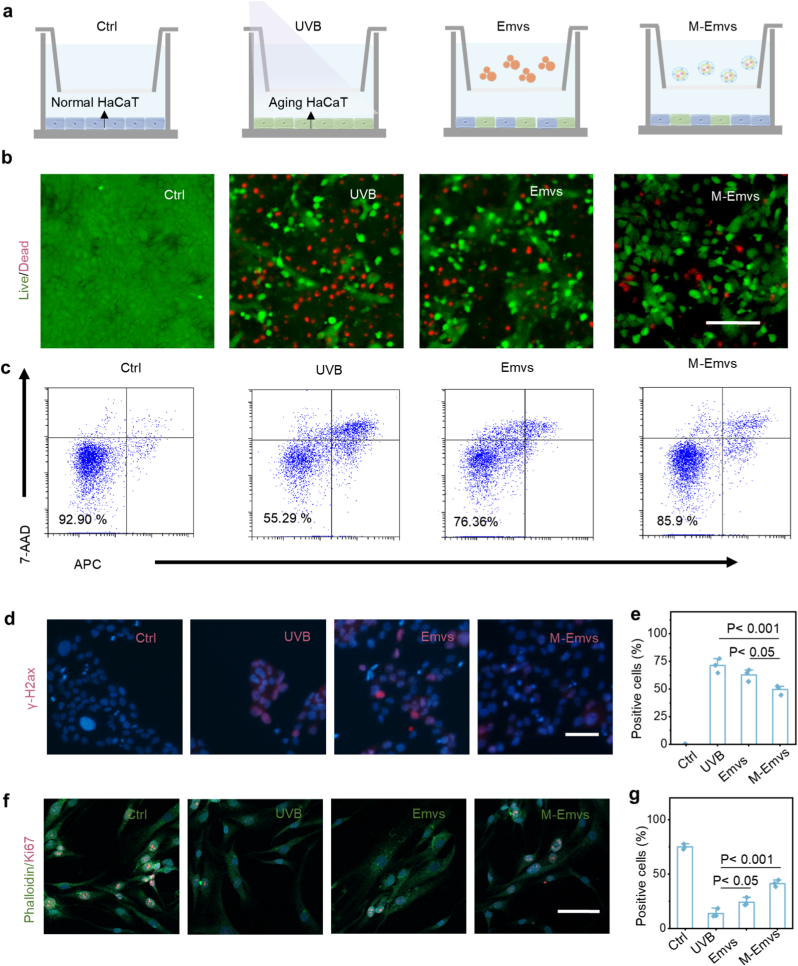
***In vitroanti*-photoaging effects of HDF-Emvs-loaded microcarriers**. a) Schematic illustration of different treatment groups. b) Representative live/dead staining images. c) Flow cytometry analysis of apoptotic cell proportion. d) γ-H2AX immunofluorescence staining (n = 3). e) Quantification of γ-H2AX-positive cell percentage (n = 3). f) Fluorescent dual staining, with green indicating the cytoskeleton and red indicating Ki67 in HDFs. g) Quantification of Ki67-positive cell percentage (n = 3). Scale bars: 100 μm (b); 50 μm (d, f). (For interpretation of the references to color in this figure legend, the reader is referred to the Web version of this article.)

One of the key mechanisms of photoaging in skin tissue is programmed cell death (apoptosis). Here, we designed an in vitro model to evaluate the *anti*-photoaging performance of the engineered microcarriers. The culture medium containing non-transfected Emvs was assigned as the Emvs group, while core–shell microcarriers with non-transfected Emvs loaded in the shell layer and transfected Emvs loaded in the core were defined as the M-Emvs group. As expected, UVB irradiation of HaCaT cells resulted in a markedly increased apoptotic ratio accompanied by pronounced morphological changes (Fig. 4a–c and Fig. S7). Treatment with both Emvs and M-Emvs significantly reduced apoptosis and overall cell death. Consistently, UVB exposure led to elevated intracellular ROS levels, whereas intervention with Emvs and M-Emvs effectively suppressed ROS accumulation (Fig. S8a–b), indicating a rapid cytoprotective and antioxidant response. Furthermore, M-Emvs treatment significantly attenuated genomic instability in HaCaT cells, as evidenced by the reduced levels of cellular senescence and DNA damage markers, including γ-H2AX (Fig. 4d and e).

In addition to the protective effect observed in the epidermis, we further investigated whether M-Emvs could promote functional recovery in the dermis. Cell viability analysis of UVB-irradiated HDF cells showed that M-Emvs treatment significantly increased fibroblast survival compared to the UVB and Emvs groups (Fig. S9a), indicating its supportive role in fibroblast resistance to UV-induced damage. To further assess the dermal regeneration potential, we evaluated the proliferation capacity of HDF cells using Ki67 staining. As shown in Fig. 4f and g, UVB irradiation significantly reduced the proportion of Ki67-positive HDF cells; however, M-Emvs treatment significantly restored Ki67 expression. Simultaneously, ELISA quantitative analysis showed that M-Emvs significantly enhanced type I procollagen secretion in UVB-damaged HDF cells (Fig. S9b), suggesting the recovery of extracellular matrix-related synthetic functions. These findings suggest that M-Emvs can effectively alleviate UVB-induced photoaging in vitro and support the successful operation of the core-shell controlled-release systems.

### 2.4 *In vivo* therapeutic effect evaluation

**Fig. 5:**
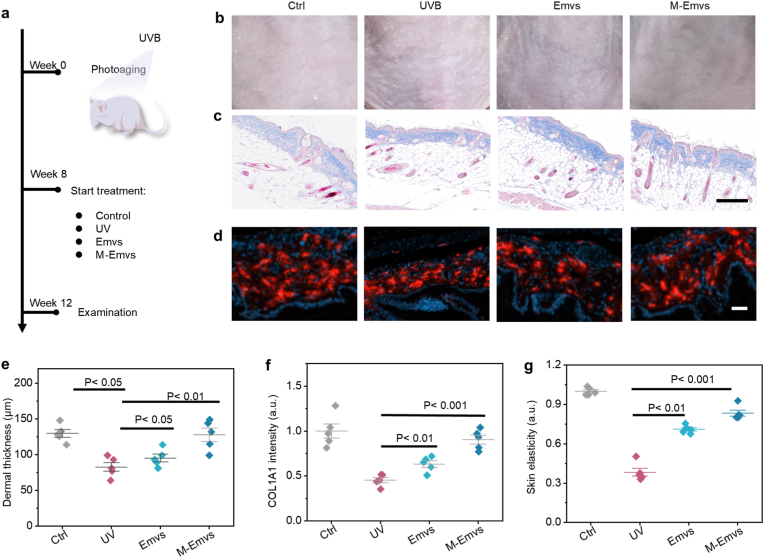
***In vivo* therapeutic effect evaluation of photodamage in an animal model.** a) Schematic illustration of the *in vivo* treatment protocol. b) Representative images of nude mouse dorsal skin in different groups at Week 12. c) Representative Masson's staining images of skin from different groups. d) Representative COL1A1 immunofluorescence staining images of skin tissue. e) Dermal thickness measurements (n = 5). f) Quantification of relative COL1A1 fluorescence intensity (n = 5). g) Relative skin elasticity scores (n = 5). Scale bars: 200 μm (c) and 50 μm (d).

The photodamage repair efficacy of different treatments was assessed in a nude mouse model over a 12-week period (Fig. 5a). At week 8, UVB-irradiated mouse skin exhibited typical features of photoaging, as shown in Fig. S10. By Week 12, the UVB-exposed group exhibited the formation of wrinkles and significant dermal thinning, indicative of severe photodamage (Fig. 5b and c). In contrast, the treatment groups exhibited notable improvements in collagen deposition (Fig. 5d). Both Emvs and M-Emvs significantly enhanced dermal structural thickness compared to the UVB group (Fig. 5e). Hematoxylin and eosin (H&E) staining was used to assess the pathological changes in the epidermis of photoaged skin. UVB irradiation caused significant epidermal thickening and structural changes, while Emvs and M-Emvs treatments alleviated these epidermal abnormalities to varying degrees, indicating that they have an ameliorative effect on UVB-induced epidermal pathological changes (Fig. S11). Collagen I deposition was markedly low in the UVB group, whereas treatment with Emvs resulted in elevated Collagen I levels to 63.3 ± 8.2% (Fig. 5f). M-Emvs treatment resulted in the most effective tissue regeneration, with Collagen I levels reaching 90.6 ± 11%. Furthermore, M-Emvs demonstrated superior efficacy over Emvs in restoring skin elasticity, as evaluated by the MPA580 multi-probe skin analysis system. The M-Emvs group showed markedly enhanced elasticity parameters relative to the UVB and Emvs groups (Fig. 5g). These results underscore the superior efficacy of M-Emvs in promoting collagen synthesis and skin repair, highlighting their potential as a novel therapeutic strategy for mitigating UVB-induced photodamage.

### 2.5 Biosafety evaluation

**Fig. 6:**
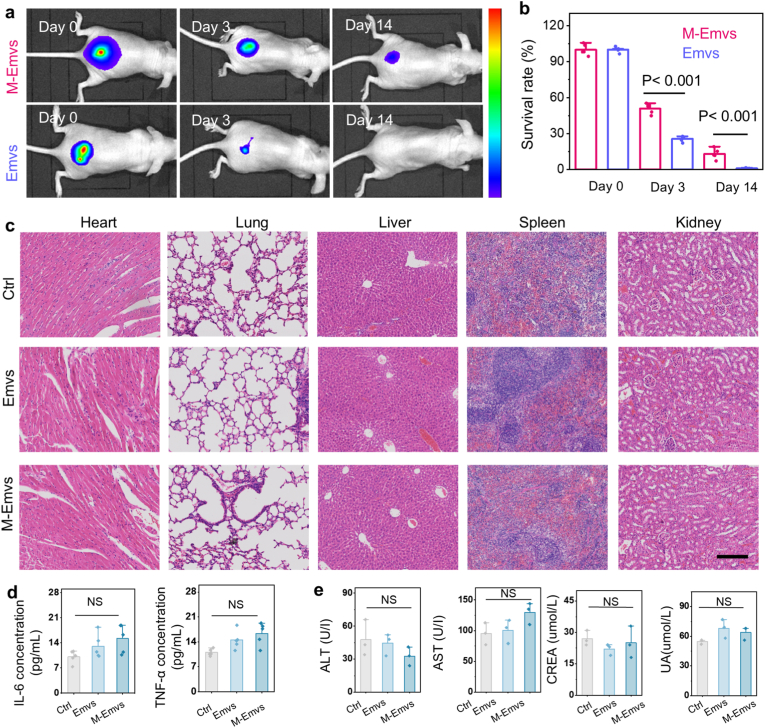
***In vivo* biosafety evaluation.** a) *In vivo* imaging of subcutaneous injection sites across different groups. b) Quantification of relative fluorescence intensity (n = 5). c) Histological analysis via H&E staining of the heart, liver, spleen, lung, and kidney tissues. d) ELISA quantification of IL-6 levels and TNF-α levels (n = 5). e) Quantification of ALT, AST, CREA, and UA levels (n = 3). The scale bar is 300 μm in c).

We monitored their distribution profiles of Emvs and M-Emvs (labelled with DiD fluorescent dye) and using live imaging after subcutaneous implantation in mice. Over 14 days, the fluorescence signal of Emvs declined significantly, faster than that of M-Emvs (Fig. 6a and b), suggesting that the microcarrier encapsulation effectively prolongs the retention and release process. For biocompatibility assessment, Emvs and M-Emvs were injected subcutaneously for 30 days, followed via hematoxylin and eosin (H&E) staining of key organs (Fig. 6c). No significant histopathological changes were observed in either group, indicating excellent biocompatibility and the absence of systemic toxicity. To investigate potential inflammatory responses, ELISA analysis was conducted to measure IL-6 and TNF-α levels in surrounding skin tissues compared to normal skin tissues (Fig. 6d). No meaningful differences were detected between groups (p > 0.05). Suggesting that neither Emvs nor M-Emvs induced notable immune reactions. Additionally, liver and kidney function were assessed (Fig. 6e). ALT and AST levels remained comparable within experiment groups (p > 0.05), indicating no hepatic damage of microcarriers. Similarly, CREA and UA levels were within the normal range, confirming no renal toxicity. These results demonstrated the excellent biocompatibility of M-Emvs.

## 3 Conclusion and prospective

We developed a novel multifunctional microcarrier system aimed at effective delivery of Emvs to treat photoaging. The M-Emvs system effectively alleviated UVB-induced photoaging by reducing apoptosis, oxidative stress, and cellular senescence, while promoting fibroblast proliferation. *In vivo* studies further confirmed that M-Emvs significantly enhanced collagen regeneration and promoted skin elasticity recovery, outperforming other treatment groups. Biosafety evaluations showed that M-Emvs exhibited excellent biocompatibility, with no evidence of systemic toxicity or severe immune activation. The microcarrier-based delivery strategy not only improved Emvs stability but also enabled controlled release, thereby maximizing therapeutic outcomes.

Despite these promising results, several limitations should be acknowledged. First, the release behavior of M-Emvs was primarily evaluated under simplified in vitro conditions, and the precise release kinetics in the complex *in vivo* skin microenvironment remain to be fully elucidated. Second, while the observed biological outcomes suggest a sequential release mechanism, direct real-time tracking of individual vesicle populations and elucidating their respective molecular mechanisms at different stages need to be performed. Additionally, the current study focused on short-to mid-term therapeutic outcomes; therefore, long-term efficacy and biodegradation behavior of the microcarrier system warrant further investigation. Future studies should aim to further optimize microcarrier properties, including tunable degradation profiles, mechanical stability, and targeted delivery capabilities. Moreover, scaling up production and conducting long-term *in vivo* evaluations will be essential steps toward potential clinical translation. Beyond dermatological applications, the ability of M-Emvs to modulate oxidative stress, apoptosis, and extracellular matrix remodeling suggests broader potential in regenerative medicine and tissue repair contexts.

## 4 Methods

**Cell culture and transfection:** Human dermal fibroblasts (HDFs, PC-202 h) purchased from SAIOS (Wuhan, China) were maintained in Dulbecco's Modified Eagle Medium (DMEM, Gibco) supplemented with 10% fetal bovine serum (FBS) and 1% penicillin–streptomycin under standard conditions (37 °C, 5% CO_2_). For electroporation, HDFs at passages three were harvested at ∼80% confluence and resuspended at a density of 1 × 10^6^ cells per reaction in the electroporation buffer supplied with the 4D-Nucleofector™ system (Lonza, Switzerland). Cells were transfected with 2 μg of COL1A1-GFP plasmid DNA (Sino Biological) using the manufacturer-recommended program. Following electroporation, cells were immediately transferred into pre-warmed complete DMEM and allowed to recover for 24 h before downstream assays.

**Emvs and exosome isolation:** Emvs were prepared using a LiposoFast LF-50 extruder (Avestin) equipped with polycarbonate microfiltration membranes of varying pore sizes (5 μm, 1 μm, and 400 nm). Briefly, transfected HDF suspensions were subjected to 10 extrusion cycles through each membrane sequentially to obtain uniform vesicles. Emvs were subsequently purified using the Total Exosome Isolation Reagent (Invitrogen, USA) according to the manufacturer's instructions. For comparison, natural exosomes (Exos) were extracted from HDF-conditioned medium via differential ultracentrifugation, consisting of sequential centrifugation at 300×*g* for 10 min, 2000×*g* for 20 min, and 10,000×*g* for 30 min to remove cells and debris, followed by ultracentrifugation at 100,000×*g* for 70 min at 4 °C (Beckman Coulter Optima XPN-100). Vesicle morphology was characterized using transmission electron microscopy (TEM) (JEM-2100), and size distribution was determined by nanoparticle tracking analysis (NanoSight NS300). Protein amounts were evaluated with the BCA assay kit from Beyotime Biotechnology (China). Particle concentration and size distribution were measured by nanoparticle tracking analysis (NTA, Malvern Panalytical, UK). Vesicle samples were diluted in filtered PBS to achieve the instrument's recommended particle count range.

**RT–qPCR analysis of vesicle-associated COL1A1 mRNA**: COL1A1 mRNA was extracted using TRIzol reagent (Invitrogen) according to the manufacturer's instructions. The isolated RNA was quantified and equal amounts of RNA were used for reverse transcription using a PrimeScript RT reagent kit (Vazyme, Nanjing, China). Quantitative PCR was performed using qPCR SYBR Green Master Mix (Yeasen, Shanghai, China) on a QuantStudio real-time PCR system (Thermo Fisher Scientific). Relative COL1A1 mRNA levels were calculated using the 2^−ΔΔCt^ method. Primer sequences used for RT–qPCR are listed in Table S1.

**Western blot analysis:** The vesicle samples were lysed using RIPA buffer containing protease inhibitors and mixed with SDS loading buffer. Proteins were separated by SDS–PAGE and transferred onto PVDF membranes (Millipore). After blocking with 5% non-fat milk in TBST for 1 h at room temperature, membranes were incubated overnight at 4 °C with primary antibodies against Alix, CD63, TSG101, and Calnexin. Subsequently, membranes were incubated with HRP-conjugated secondary antibodies for 1 h at room temperature. Protein bands were visualized using an enhanced chemiluminescence (ECL) detection system (Thermo Fisher Scientific).

**Stability of HDF-Emvs:** To assess serum stability, HDF-Emvs were incubated in serum-containing medium (or serum solution) at room temperature or 4 °C for the indicated time points. At each time point, aliquots were collected and immediately analyzed by NTA. For proliferation assay, un-transfected HDFs were seeded in 96-well plates and treated with increasing concentrations of COL1A1-Emvs for 72 h. Cell proliferation was assessed using CCK8 according to the manufacturer's instructions, and signals were normalized to the control group. Cell culture supernatants were collected after treatment. The media were centrifuged at 300×*g* for 5 min to remove cell debris, and the clarified supernatants were analyzed immediately. The concentration of human type I procollagen was measured using a human type I procollagen ELISA kit (Elabscience Biotechnology, China) according to the manufacturer's instructions.

**Microcarrier fabrication and characterization:** Microcarriers were fabricated using a microfluidic electrospray system composed of two coaxially aligned glass capillaries fixed on a microscope slide. The outer phase consisted of 2% (w/v) medium-viscosity sodium alginate, while the inner phase was prepared by mixing recombinant human collagen methacrylate (RHCMA, 15 %). Under a high-voltage electric field, the coaxial flow was disrupted into uniform core–shell droplets, subsequently collected in a calcium chloride solution to quickly promote ionic crosslinking of the alginate shell. After collection, the inner core was subsequently stabilized via ultraviolet (UV) crosslinking (8 mW/cm^2^, 5 min), resulting in structurally robust and well-defined core–shell microcarriers suitable for downstream applications. The size distribution and surface morphology were analyzed using ImageJ and SEM, respectively. Fluorescence labeling of the core and shell phases further verified the spatial segregation of components within the microcarriers.

**Vesicle encapsulation efficiency:** The encapsulation efficiency (EE) of vesicles within the microcarriers was quantified by measuring the non-encapsulated particles using Nanoparticle Tracking Analysis (NTA). Briefly, vesicles were suspended in the alginate (shell phase) and RHCMA (core phase) precursor solutions, respectively. The total particle number of vesicles initially introduced (N_input_) and that detected in the washing supernatant (N_supernatant_) were calculated from the particle concentration determined by NTA and the corresponding sample volumes. Encapsulation efficiency was calculated as follows: EE (%) = (N_input_ − N_supernatant_)/N_input_ × 100%.

**Release behavior study:** The release kinetics of model fluorescent nanoparticles were evaluated to characterize the controlled-release behavior of the core–shell microcarriers. Nanoparticle-loaded microcarriers were suspended in phosphate-buffered saline (PBS, pH 7.4) and incubated at 37 °C under gentle shaking. At predetermined time points, samples were collected and centrifuged, and the supernatant was analyzed by measuring fluorescence intensity to quantify the released nanoparticles. An equal volume of fresh buffer was replenished after each sampling. The cumulative release was calculated and expressed as the percentage of released nanoparticles relative to the total encapsulated amount.

***In vitro* UVB-induced photoaging model:** HaCaT keratinocytes were exposed to UVB (311 nm) irradiation for 20 min to establish an in vitro photoaging model. The UVB lamp was positioned 20 cm above the surface of the culture plate. Cell apoptosis and morphological changes were evaluated using flow cytometry and fluorescence microscopy, respectively. Intracellular ROS levels were assessed using the DCFH-DA fluorescent probe kit (Beyotime, China). Cellular senescence was further assessed by senescence-associated β-galactosidase (SA-β-gal) staining using a β-galactosidase staining kit (Beyotime, China). DNA damage was analyzed by γ-H2AX immunofluorescence staining (Thermo Fisher Scientific, USA). For primary human dermal fibroblasts (HDFs), the UVB-induced photoaging model was established under the same irradiation conditions as described above. Cell survival was further evaluated using a trypan blue exclusion assay. Collagen synthesis was evaluated by measuring secreted type I procollagen levels in the culture supernatant using a Human Procollagen I ELISA kit, following the manufacturer's instructions. All experiments were performed with at least three independent biological replicates.

***In vivo* photodamage study:** Animals were randomly assigned to the experimental groups using a simple randomization method. All assessments were performed in a blinded manner to minimize potential bias. A UVB-induced photoaging mouse model was established by exposing nude mice to UVB radiation (Philips, 311 nm) at a distance of 15 cm. Mice (n = 5) were then treated with MOP-8 (0.1 mg/ml) every two days over a period of eight weeks. The mice were randomly assigned to four groups: Ctrl, UVB, Emvs-treated, and M-Emvs-treated. After the 8-week irradiation period, treatments were administered via subcutaneous injection. Skin morphology and collagen deposition were evaluated by H&E and Masson's trichrome staining (Beyotime Biotechnology, China) and COL1A1 immunofluorescence staining (Thermo Fisher Scientific, USA). Skin elasticity was quantitatively measured using a Cutometer® MPA580 system (Courage + Khazaka Electronic GmbH, Germany). Animal experiments were approved by University of Chinese Academy of Sciences of Wenzhou Institute (WIUCAS24122309).

**Biosafety evaluation:** To evaluate biodegradability, Emvs and M-Emvs were subcutaneously implanted into the dorsal region of nude mice, and the *in vivo* retention was tracked using live fluorescence imaging (IVIS Spectrum, PerkinElmer, USA) over a period of 14 days. Fluorescence intensity was quantified. At 30 days post-implantation, major organs were collected for histopathological examination via hematoxylin and eosin (H&E) staining. Histological sections were imaged using a microscope (Leica DM2500, Germany), and tissue morphology was analyzed. Systemic toxicity was assessed by a fully automated biochemical analyzer (Cobas c311, Switzerland). Interleukin-6 and tumor necrosis factor-alpha concentrations were determined via ELISA kits (antibodies-online, Germany) following the manufacturer's protocols, with absorbance readings taken on a microplate reader (Synergy H1, USA).

**Statistical Analysis:** All quantitative data are presented as mean ± SEM from at least three independent biological replicates. Statistical significance was assessed using Student's t-test, with p < 0.05 considered significant. All experiments were performed with at least three independent replicates.

## CRediT authorship contribution statement

**Xiang Lin:** Data curation, Methodology, Writing – original draft. **Anne M. Filppula:** Writing – review & editing. **Luoran Shang:** Writing – review & editing. **Hongbo Zhang:** Supervision, Writing – review & editing. **Dexuan Wang:** Supervision, Writing – review & editing.

## Declaration of competing interest

The authors declare that they have no known competing financial interests or personal relationships that could have appeared to influence the work reported in this paper.

## Data Availability

Data will be made available on request.
