# Supplementary material for: Microcarriers encapsulating COL1A1 mRNA-loaded nanovesicles for skin photoaging treatment
Source: Mater Today Bio. 2026 Apr 15;38:103126. doi: 10.1016/j.mtbio.2026.103126 (PMC13101773; doi:10.1016/j.mtbio.2026.103126)
Supplement: Multimedia component 1 [file mmc1.docx]

**Microcarriers Encapsulating COL1A1 mRNA-loaded Nanovesicles for Skin Photoaging treatment**

Xiang Lin ^1,2^, Anne M. Filppula^2^, Luoran Shang ^3,^**,* Hongbo Zhang ^2,^*, Dexuan Wang ^1,^*

1. Department of Pediatrics, The Second Affiliated Hospital and Yuying Children's Hospital of Wenzhou Medical University, Wenzhou, 325027, China
2. Pharmaceutical Sciences Laboratory, Åbo Akademi University, Turku 20520, Finland
3. Shanghai Xuhui Central Hospital, Zhongshan-Xuhui Hospital, and the Shanghai Key Laboratory of Medical Epigenetics, International Co-laboratory of Medical Epigenetics and Metabolism (Ministry of Science and Technology, Institutes of Biomedical Sciences), Fudan University, Shanghai 200032, China

Email: [wangdexuan@wmu.edu.cn](mailto:wangdexuan@wmu.edu.cn) (D.X.W), [luoranshang@fudan.edu.cn](mailto:luoranshang@fudan.edu.cn) (L.R.S); [hongbo.zhang@abo.fi](mailto:hongbo.zhang@abo.fi) (H.B.Z)

**Supporting Figures:**


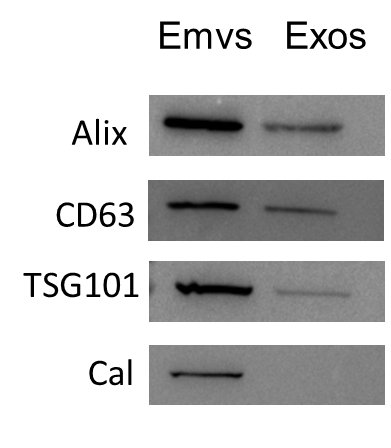


**Figure S1.** Representative western blot images showing the expression of associated protein markers in extrusion-derived membrane vesicles (Emvs) and naturally secreted exosomes (Exos).


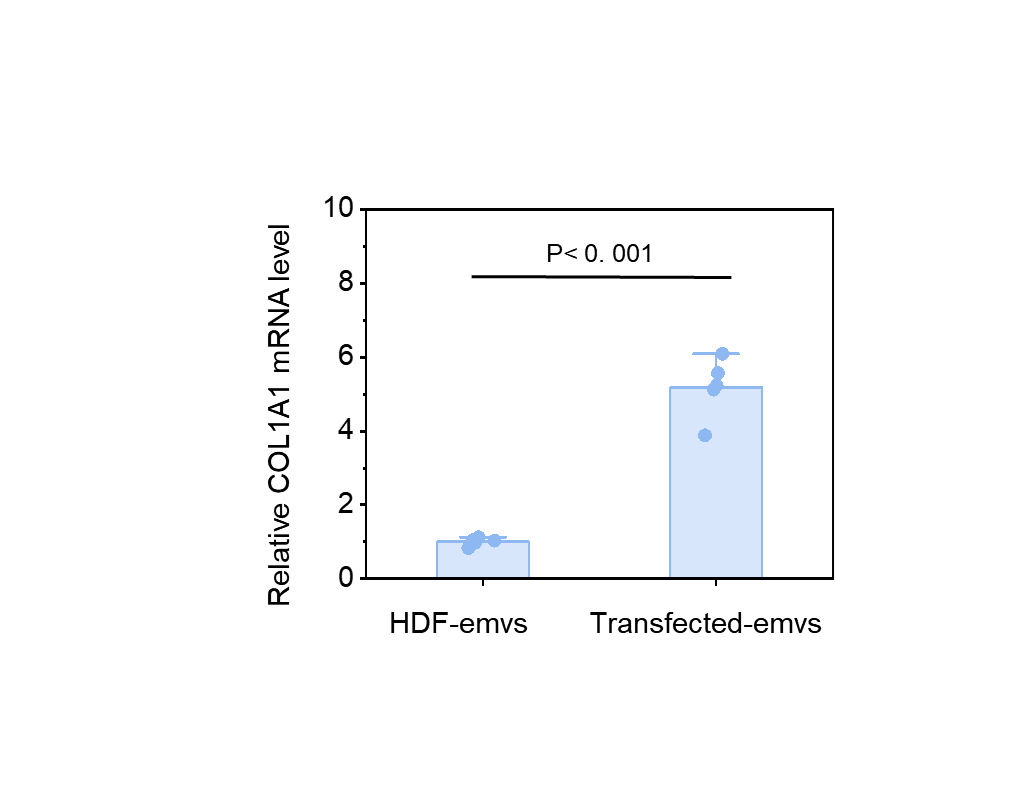


**Figure S2.** Relative COL1A1 mRNA loading per vesicle. COL1A1 mRNA levels were quantified by RT–qPCR, normalized to the HDF-exos group.


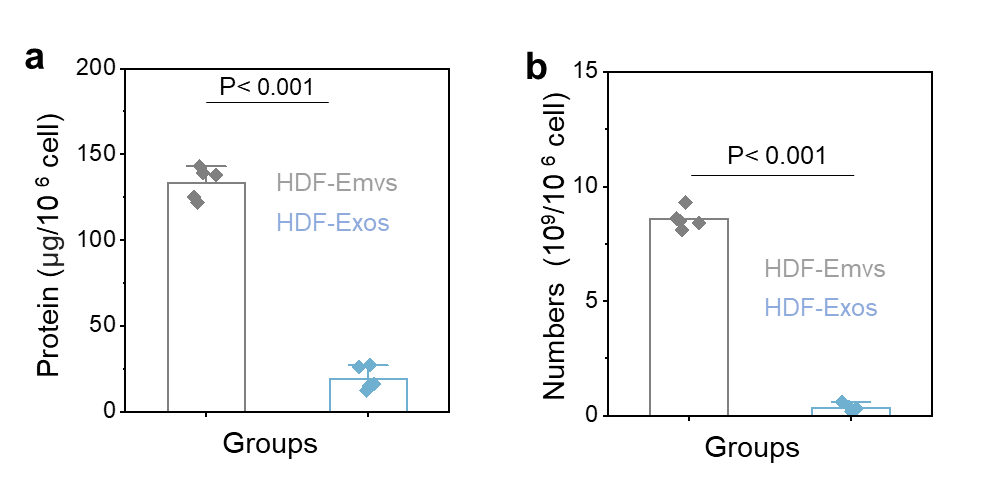


**Figure S3.** a) Total protein content in HDF-Emvs and HDF-Exos. b) Total particle numbers in HDF-Emvs and HDF-Exos.

**
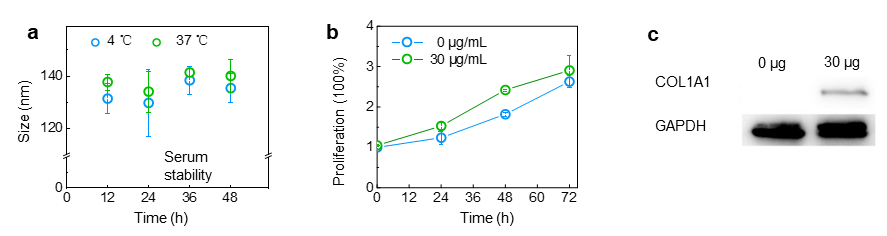
**

**Figure S4.** a) Stability of HDF-Emvs in serum. b) Proliferative effect of COL1A1-Emvs on HDFs. c) Western blot analysis of canonical vesicle markers.

**
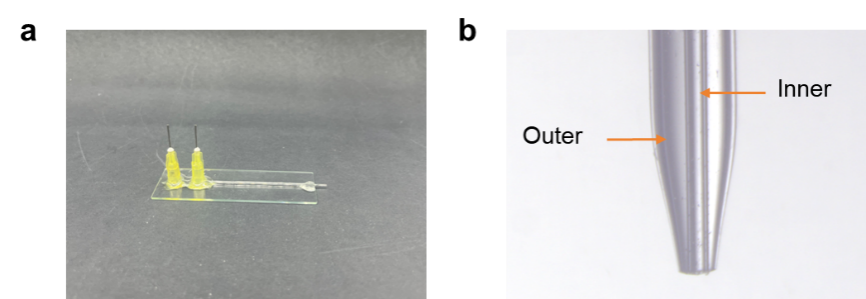
**

**Figure S5.** a) Photograph of the device for generating microcarriers. b) Microscopic image of the assembled capillary tip.

**
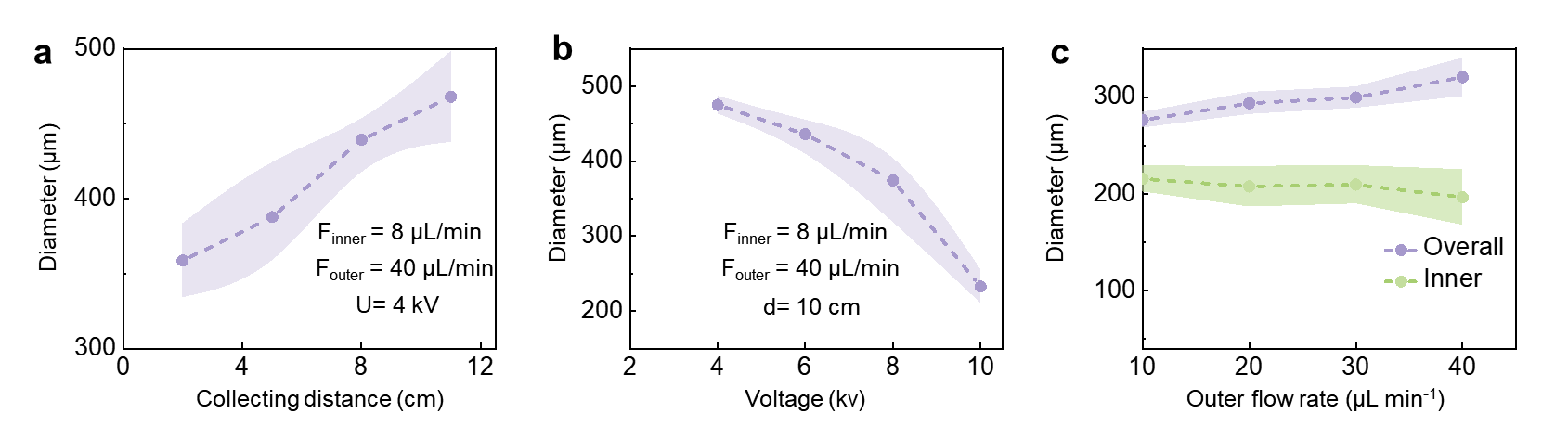
**

**Figure S6.** a) Effect of collecting distance on microcarrier diameter. b) Effect of voltage on microcarrier diameter. c) Effect of external phase flow rate on the core diameter and overall diameter.


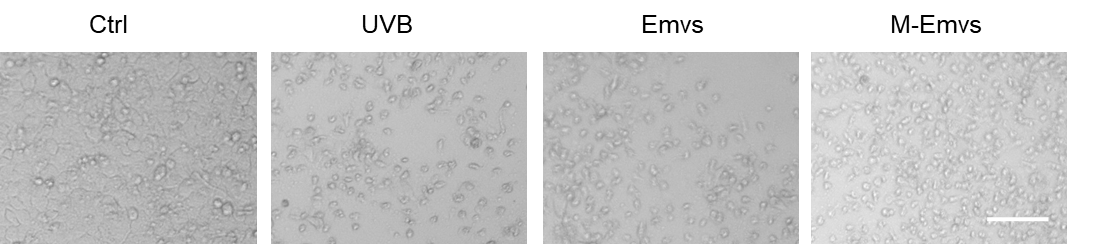


**Figure S7.** Microscope images of HaCaT cells after different treatments. The scale bar is 100 μm.

**
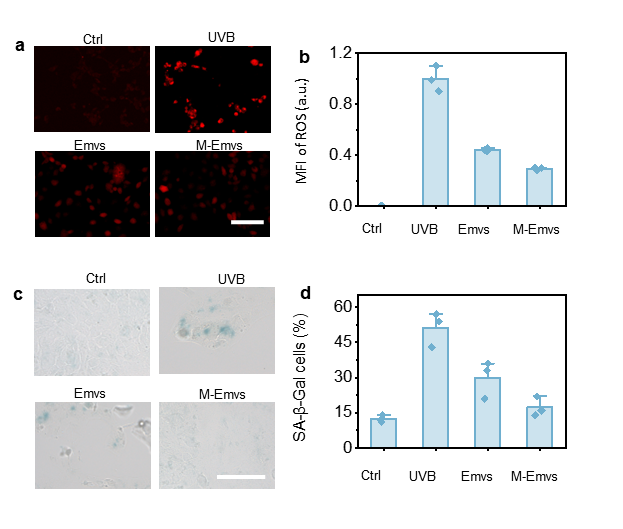
**

**Figure S8.** a) Fluorescence images of ROS content in HaCaT cells after different treatments. The fluorescence signal was pseudocolored in red during image processing. b) Mean fluorescence intensity (MFI) of ROS. (c) Senescence-associated β-galactosidase (SA-β-Gal) staining of UVB-exposed cells after different treatments, with representative bright-field images and corresponding quantitative analysis of (d) SA-β-Gal–positive cells. The scale bar is 50 μm in a) and c).


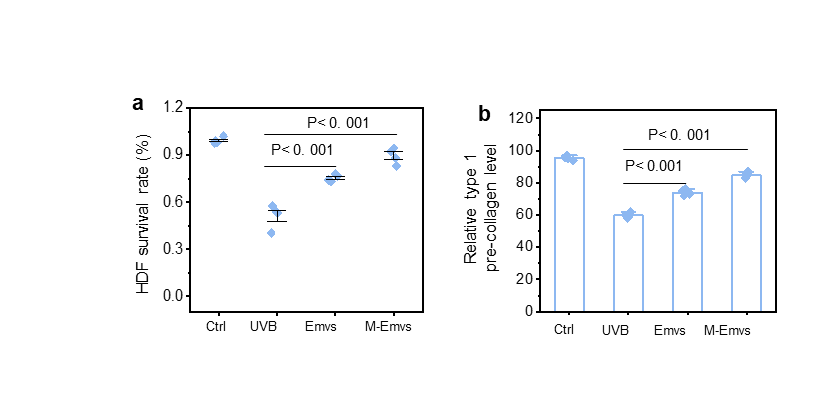


**Figure S9.** (a) Quantitative analysis of cell viability for the control, UVB, UVB + Exos, and UVB + M-Emvs groups. (b) Relative type 1 pre- collagen level in different groups.


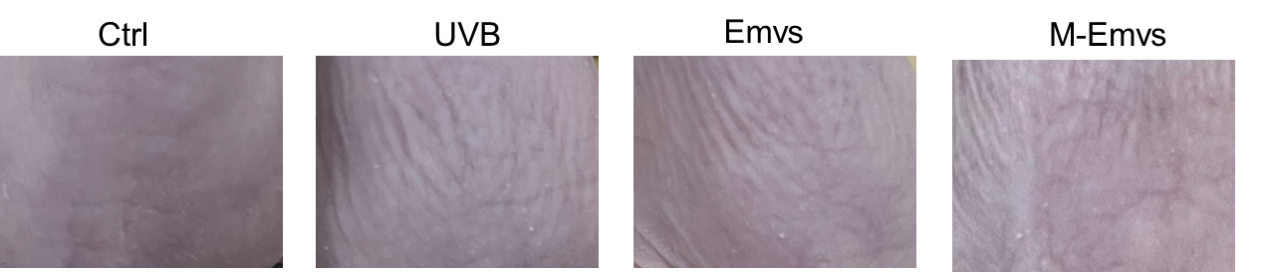


**Figure S10**. Representative images of nude mouse dorsal skin in different groups at Week 8.


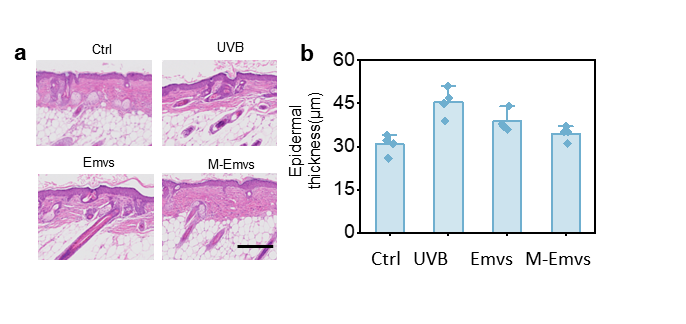


**Figure S11.** (a) Representative H&E-stained sections of skin tissue from the Control, UVB, UVB + Exos, and UVB + M-Emvs groups. (b) Epidermal thickness was quantified from H&E images (n = 5). The scale bar is 10 μm.

**Table S1.** Primers designed for mRNA detection

| **Gene name** | **Primer** |
| --- | --- |
| Human GAPDH F | CAGCCTCAAGATCATCAGCA |
| Human GAPDH R | AGAGGCAGGGATGATGTTCT |
| COL1A1-GFP F | CCTGGAAAGAATGGAGATGA |
| COL1A1-GFP R | ACCATCCAAACCACTGAAAC |
